# Ag-YOLO: A Real-Time Low-Cost Detector for Precise Spraying With Case Study of Palms

**DOI:** 10.3389/fpls.2021.753603

**Published:** 2021-12-24

**Authors:** Zhenwang Qin, Wensheng Wang, Karl-Heinz Dammer, Leifeng Guo, Zhen Cao

**Affiliations:** ^1^Agricultural Information Institute, Chinese Academy of Agricultural Sciences, Beijing, China; ^2^Leibniz Institute for Agricultural Engineering and Bioeconomy, Department Engineering for Crop Production, Potsdam, Germany

**Keywords:** object detection, precise spraying, embedded AI, YOLO, NCS2

## Abstract

To date, unmanned aerial vehicles (UAVs), commonly known as drones, have been widely used in precision agriculture (PA) for crop monitoring and crop spraying, allowing farmers to increase the efficiency of the farming process, meanwhile reducing environmental impact. However, to spray pesticides effectively and safely to the trees in small fields or rugged environments, such as mountain areas, is still an open question. To bridge this gap, in this study, an onboard computer vision (CV) component for UAVs is developed. The system is low-cost, flexible, and energy-effective. It consists of two parts, the hardware part is an Intel Neural Compute Stick 2 (NCS2), and the software part is an object detection algorithm named the Ag-YOLO. The NCS2 is 18 grams in weight, 1.5 watts in energy consumption, and costs about $66. The proposed model Ag-YOLO is inspired by You Only Look Once (YOLO), trained and tested with aerial images of areca plantations, and shows high accuracy (F1 score = 0.9205) and high speed [36.5 frames per second (fps)] on the target hardware. Compared to YOLOv3-Tiny, Ag-YOLO is 2× faster while using 12× fewer parameters. Based on this study, crop monitoring and crop spraying can be synchronized into one process, so that smart and precise spraying can be performed.

## 1. Introduction

### 1.1. Motivation and Background

*Areca catechu L*. is also known as betel palm. It is cultivated mainly in tropical areas as South East Asia, India, South Pacific, and some African and Caribbean regions (Heatubun et al., 2012). The seed (areca nut) harvested is chewed in most cases because of the stimulating effect of its alkaloids. In a word, it is an importantly high-value crop. In the Hainan Island of China, this crop provides a livelihood to more than 2 million people in rural areas. Unfortunately, this cultivar has been suffering from the yellow leaf disease (YLD) that may lead to the decay and wilt of the palms. Luo et al. (2001) employed various methods to prove that the areca YLD in Hainan is an infectious disease caused by phytoplasma, including electron microscope observation, tetracycline antibiotic injection diagnosis, Polymerase Chain Reaction (PCR) technology. According to the studies, pests play a vital role in the spreading of viruses and phytoplasma. Therefore, spraying pesticides on the palms constantly is an effective way to prevent YLD from spreading.

With the advent of the Internet of Things (IoT), especially the rapid evolution of the UAV technology, combined with image data analytics, PA technologies have been developed, to increase productivity and at the same time reduce environmental impact. The PA technology focuses on about 20 relevant applications (Radoglou-Grammatikis et al., 2020), among which aerial monitoring and crop spraying are the most common. Spraying UAVs carry different types of equipment developed (Xiongkui et al., 2017; Lan and Chen, 2018; Yang et al., 2018) to spray pesticides to the crops in small fields or rugged environments, such as mountain areas. However, since an aerial crop monitoring process has not been synchronized with the spraying platform, today's UAVs spray the entire area uniformly with pesticides. Safe and effective spraying must be performed in areca protection due to the fact that: 1) In areca plantations, there is a certain distance from one palm to another; 2) Pesticides can have several side effects on the biotic and abiotic environment and bear a risk to harm human health (Horrigan et al., 2002); 3) Palms are irregular, especially in height, which increases the risk of UAV crashes.

For this purpose, this study develops an onboard CV component for spraying UAVs. The system takes RGB data acquired by the low-cost onboard camera as input, inferences and then sends instructions to the flight management unit (FMU) of the UAV in a real-time manner. As a result, pesticides can be applied on a per-plant basis, with a variable dosage subject to the severity of plant diseases. To be specific, the key feature of this system is to perform object detection in real-time.

In object detection tasks, deep learning (DL) methods significantly outperform other existing approaches due to their robustness to the diversity of targets. Nevertheless, the powerful performance of DL often comes with a high computation complexity and intensive memory demand, mainly required by the convolutional layers in convolutional neural network (CNN). For a high-end Graphics Processing Unit (GPU), this is not a problem. However, UAVs are tight constraints in computational power, memory size, and energy consumption. We solve the issue by extending the computing capacity with embedded hardware, then developing a new algorithm to fit. Embedded AI computing options are investigated, including graphics processing units, vision processing units, and field-programmable gate arrays. Today, commercial products are on the market, such as NVIDIA (https://www.nvidia.com/), Intel (https://www.intel.com), and MYiR (http://www.myir-tech.com/). As listed in Table 1, Intel NCS2 has both the least weight and power consumption. Besides, for a battery-powered device, those features are of great advantage.

**Table 1:** Main specifications of the candidate platforms in this study.

	**Intel NCS2**	**nVidia Jetson Nano**	**MYiR ZU3EG**
Features size	73 × 36 mm	70 × 45 mm	100 × 70 mm
HW accelerator	Myriad X VPU	128-core nVidia Maxcell GPU	Xilinx ultraScale
CPU	N./A.	Arm A57	MPSoC XCZU3EG (4-core Arm A53)
Peak performance	150 GFLOPs	472 GFLOPs	1.2 TFLOPs
Data precision	FP16	FP16/FP32	FP32
Nominal power	1.5 W	10 W	10 W
Weight	18 g^*^	140 g	150 g

* *Weight of NCS2 is not including the outer shell*.

### 1.2. Scope and Contribution

The overall goal of this study is to develop an object detection algorithm, which can run on NCS2 in real-time. We study the efficient object detection algorithms optimized for resources-constraint hardware and propose a novel model, as it is derived from the famous YOLO (Redmon et al., 2016), and used for agricultural purposes. Hence, we call it Ag-YOLO. Specifically, the summary of the contributions presented in this study is the following:

- We provide a thorough, complete description of the design, deployment, and assessment of an intelligent real-time agricultural object detection system based on embedded AI.
- By proposing the Ag-YOLO object detection algorithm and testing it on the NCS2, we demonstrate that a DL-based CV algorithm can be implemented on resource-constraint hardware, to deal with real-life PA challenges. On the most cost-efficient embedded AI device, the NCS2, our Ag-YOLO can achieve 36.5 fps with satisfying accuracy. The accuracy of Ag-YOLO is always higher than YOLOv3-Tiny in different input dimensions, and the highest accuracy of Ag-YOLO is 0.7655.
- We demonstrate a whole process to build an efficient object detector for palms. This method is easy to propagate to other cash crops such as pitaya, citrus.
- We developed a tool that is used for data training and transforming PC models to the NCS2 platform.
- We propose the "channel reorganization" block to adapt the ShuffleNet-v2 (Ma et al., 2018) backbone to NCS2, which shows the best speed performance.

### 1.3. Article Structure

The remainder of the study is organized as follows: Section 2 reviews related works on smart UAVs and Embedding AI. The proposed Ag-YOLO in this study is presented in Section 3. The experimental results, as well as a comparison to the baseline categorization and discussion, are presented in Section 4. Finally, Section 5 provides a summary of the study.

## 2. Related Work

In this section, we review vision-based smart UAV applications. Furthermore, efficient embedded object detection algorithms are then discussed. Finally, works based on improved YOLO are presented.

### 2.1. Vision-Based Smart UAV Applications

In recent studies of UAVs, Intel NCS2 (https://www.intel.com/content/www/us/en/developer/tools/neural-compute-stick/overview.html), NVIDIA Jetson Nano (https://developer.nvidia.com/embedded/jetson-nano), NVIDIA Jetson TX2 (https://developer.nvidia.com/embedded/jetson-tx2) are used as companion computers in vision-based smart UAV applications to process aerial imagery. Then the output result is used to control the UAV's FMU. Dobrea and Dobrea (2020) places two embedded companion computers, a Raspberry Pi (RPi) and a Jetson Nano, on a HoverGames quadcopter to follow a pre-programmed flight route and simultaneously detect humans as well as of warning the system operator to reinforce the quarantine zones for epidemic prevention purposes. In the field of early fire detection and alarm, Nguyen et al. (2021) implements a real-time fire detection solution for vast area surveillance using the UAV with an integrated visual detection and alarm system. The system includes a low-cost camera, a lightweight companion computer, a flight controller, and localization-and-telemetry modules. A Jetson Nano is used to support real-time detection, achieving a speed of 26 fps. In Afifi et al. (2019), the authors built a robust, real-time pedestrian detection system on Jetson TX2 for monitoring pedestrians by a UAV. Barisic et al. (2019) built a vision-based system for real-time detection and following of UAVs. The system achieves a real-time performance of 20 fps. Earlier, in Rabah et al. (2018), a small CPU RPi is used. In Alsalam et al. (2017), the authors developed an autonomous UAV using an Odroid U3+ and ROS to fulfill vision-based onboard decision making for remote sensing (RS) and PA.

### 2.2. Efficient Object Detection Algorithms

To detect objects in real-time with an embedded device, an efficient algorithm is required. DL-based object detection technology, which has rapidly developed since the mid-2000s, has overcome the limitations of the performance of other existing technologies, and their capabilities are similar to those of humans or sometimes exceed human abilities.

Among all the DL-based object detection frameworks, the YOLO-series (Redmon et al., 2016; Redmon and Farhadi, 2017; Farhadi and Redmon, 2018; Bochkovskiy et al., 2020) are widely used in various applications based on object detection in recent years due to their outstanding performance in terms of latency. In addition, the YOLO series algorithms also provide a trade-off between speed and accuracy, which allows researchers to apply them in different scenarios.

Although YOLOv4 (Bochkovskiy et al., 2020) has been released recently, YOLOv4 does not make any revolutionary improvement in architecture aspect to its forefather. YOLOv3 is still one of the most widely used detectors in the industry due to the limited computation resources and the insufficient software support in various practical applications. Hence, we choose YOLOv3 (Farhadi and Redmon, 2018) as our starting point and adopt some “Bag of freebies” strategies from YOLOv4. Specifically, the model we choose is the “lighter” version of YOLOv3, called Tiny-YOLOv3, which was designed with speed in mind and is generally reported as one of the better performing models in the aspect of speed and accuracy trade-off.

A YOLO-family detector is composed of backbone, neck, and head. The backbone is responsible for feature extraction, the neck synthesizes the features from backbone, and the head classifies the objects and labels the bounding boxes. As for the backbone part, there have been rising interests in improving it to achieve better speed in embedded devices, such as Howard et al. (2017); Ma et al. (2018); Wang et al. (2018), and Zhang et al. (2018). We investigate the effect by replacing them in YOLOv3-Tiny in the next section.

### 2.3. Improved Work Based on YOLO

Huang et al. (2018) proposed YOLO-Lite for bringing object detection to non-GPU computers. YOLO-Lite achieved 21 fps on a non-GPU computer and 10 fps after being implemented onto a website with only 7 layers and 482 million FLOPS. This speed is 3.8× faster than SSD Mobilenetv1, the fastest state-of-the- art model at that time. However, performances on embedding systems were not investigated.

Kim et al. (2020) investigated the performance degradation of spiking neural networks (SNNs) and presented the first spiked-based object detection model, called Spiking-YOLO. Spiking-YOLO achieves remarkable results that are comparable (up to 98%) to those of Tiny-YOLO on non-trivial datasets, PASCAL VOC, and MS COCO. Furthermore, Spiking-YOLO on a neuromorphic chip consumes approximately 280 times less energy than Tiny-YOLO and converges 2.3–4 times faster than previous SNN conversion methods.

Wong et al. (2019) introduced YOLO Nano, a highly compact deep CNN for embedded object detection designed using a human-machine collaborative design strategy, running on a Jetson AGX Xavier embedded module at different power budgets. At 15 and 30 W power budgets, YOLO Nano achieved inference speeds of ~ 26.9 and ~ 48.2 fps, respectively. The model size of YOLO Nano was 4.0 MB, which is 15.1× smaller than Tiny YOLOv2 and 8.3× smaller than Tiny YOLOv3. Despite being much smaller in model size, it achieved an mAP of 69.1% on the VOC 2007 test dataset, which is ~ 12 and ~ 10.7% higher than that of Tiny YOLOv2 and Tiny YOLOv3, respectively. Jetson AGX Xavier is a high-end embedded device, which is not considered in this study.

xYOLO is proposed in Barry et al. (2019) to detect balls and goal posts at ~ 10 fps, on a piece of low-end hardware, the RPi 3 B, in a RoboCup Humanoid Soccer competition, compared to Tiny-YOLO which achieved 0.14 fps.

Hurtik et al. (2020) presented Poly-YOLO, which improves YOLOv3 in three aspects. It is more precise, faster, and able to realize instance segmentation. Poly-YOLO has only 60% of parameters of the YOLOv3 but improves the accuracy by a relative 40%.

In Pham et al. (2020), Minh-Tan Pham et. al, designed YOLO-fine which is based on the state-of-the-art YOLOv3 with the main purpose of increasing the detection accuracy for small objects while being light and fast to enable real-time prediction within further operational contexts, providing the best compromise between detection accuracy (highest mAP), network size (smallest weight size), and prediction time (able to perform real-time prediction). No latency data are provided in this study.

## 3. Materials and Methods

To the best of our knowledge, there is no public datasets for palms available, so we build one for this study.

### 3.1. Dataset

**Figure 1:**
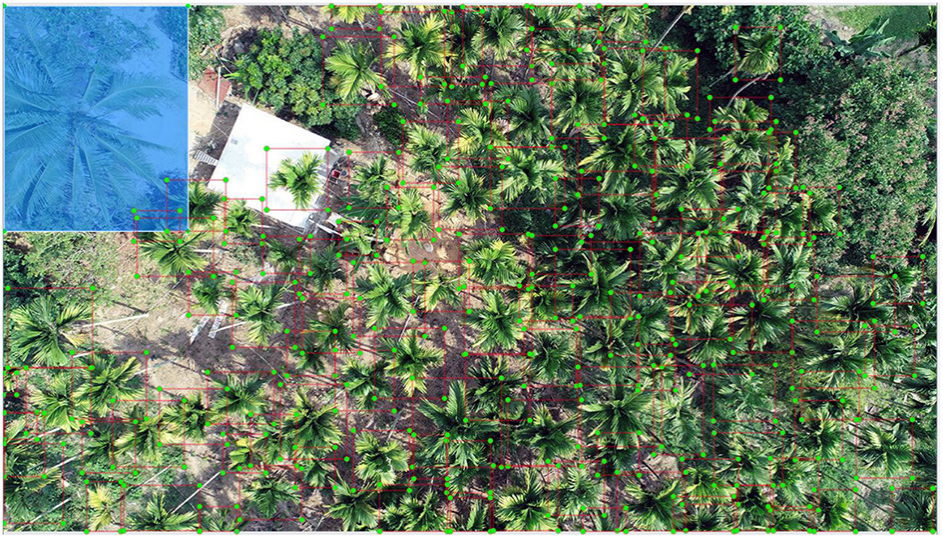
Areca palm plantation at the experimental site labeled with red quadrates. The blue semitransparent box covers a coconut palm (part of the background).

Four experimental sites in Sanya (18°15′10”N 109°30′42” E)^1^ were selected to collect aerial images. In the experimental sites, areca palms of different ages were grown (from 2 years old to more than 20 years old). In addition, their spatial distance from palm to palm varies too. Some plantations were also heterogeneous in terms of the individual trunk volume. Therefore, a high object variation was guaranteed. Sanya is a city on the Hainan Island of China. The images were collected by using a DJI Phantom 4,^2^ of which the camera resolution was 5,472 × 3,078, and the aperture was F 1/2.8. This UAV hovered above the palms at a height ranging from 2 to 10 m to take photos so that images could be in different scales. The angle of view of the camera was between 45 and 90°. An example image is shown in Figure 1 (For better illustration, this image was taken from a much higher altitude for readers who have no idea of what an areca plantation is like). More than 1,000 aerial images were taken at different times on August 2, August 3, October 5, and November 4, 2018. The collecting time ranges from early morning, midday to sunset to get different sunlight conditions. Among them, 400 images were picked and then resized to a smaller dimension (the maximum length was set to 1,500 to speed up the processing time), which were labeled with an open-source software named labelImg.^3^ After that, 300 images were randomly selected and saved into the “Training” dataset and the rest 100 images were saved into the “Testing” dataset.

It is worth noting, there are two reasons we did not use all 1,000 images. First, it took a lot of manually repetitive labor to label images; Second, with “Data Augmentation” and “Hard Negative Mining” technologies, for a small model which only detects objects of one class, 400 images are enough.

During training, we used the “Data Augmentation” method to expand the training dataset. A labeled image was cropped randomly and resized to the dimension of the network (e.g., 416 × 416). Then, it was transformed into the HSV (Hue, Saturation, and Value) color space, so that any of the H, S, and V values could be adjusted randomly for the simulation of illumination changes, or color changes. For example, reducing the H value by 1 or 2 to simulate a little less illumination intensity. After that, the image was transformed back to RGB (Red, Greed, and Blue) color space. This was implemented by using the tool *RQNet*, and the default parameter values for “Data Augmentation” are used.

### 3.2. Data Training

All frameworks were trained on an end-to-end basis in a single T1060 GPU optimized by Adam (Kingma and Ba, 2014) at the initial learning rate of 0.001. Each mini-batch has 10 images. Therefore, one epoch includes 15 mini-batches. This study resized the input dimension to (352, 352), (384, 384), (416, 416), (448, 448), (480, 480), (512, 512), (544, 544), and (576, 576) for every epoch randomly.

For every model, the parameters were initialized by the Xavier method. After using strong data augmentation, we found that ImageNet pre-training is no more beneficial, we, thus, train all the following models from scratch. By adopting Leaky ReLU as an activation function and using Gaussian distribution initialized parameter, all the models were easily converged in hundreds of thousands of iterations, taking 2 ~ 4 days on an ASUS TUF Gaming FX86FM laptop. The value of Gaussian parameters μ = 0, σ = (16*n*)^−0.5^ where *n* refers to the number of weight elements.

#### 3.2.1. L2-Norm Regularization and NCS2 Deployment

Regularization has been introduced into DL for a long time, which brings in additional information for the prevention of over-fitting. The L2-norm regularization can be expressed as follows:


(1) $$\begin{array}{l} L=\underset{\left(x,y\right)}{\sum }l\left(f\left(x,W\right),y\right)+\lambda \underset{w}{\sum }w^{2} \end{array}$$

where λ refers to the super parameter, and in YOLO articles, it is referred to as “decay” or “weight decay,” and set to 0.0005. The *x* and *y* denote the coordinate value of the feature map, and *w* denotes the parameters of the model.

**Figure 2:**
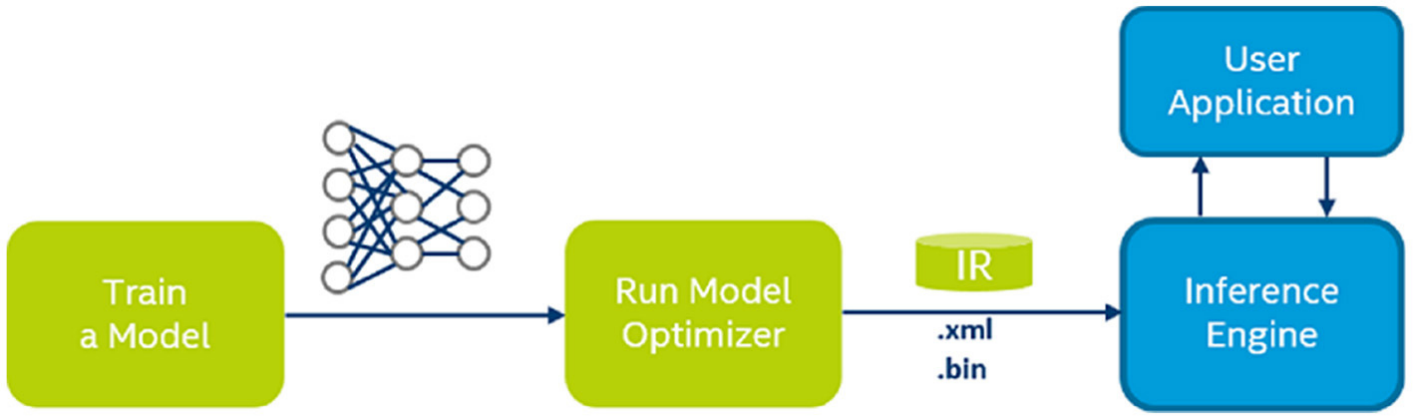
Network training and deployment on Neural Compute Stick 2 (NCS2).

In this study, the model was trained on a PC, and tested and used on NCS2, as described in Figure 2. The well-trained model on a PC is composed of a large number of parameters in FP32 format. They need to be parsed and converted to FP16 format and organized in a form that the NCS2 driver can understand.

Two pieces of C++ tools have been developed for data training and model testing on NCS2, the *RQNet*, and the *OpenVINOMyriad* (refer to https://github.com/rossqin/). The *RQNet* was used for data training and model evaluation under the Windows operating system, which was also used to convert models used by the *OpenVINOMyriad* to run on NCS2. During the training phase, the CUDA 11.1 and cuDNN 8.0 libraries were used, and all the parameters were in standard 32-bit float point values (FP32). However, NCS2 only supports 16-bit “half precision” float point values (FP16), which can express values within the range ±65,504 with the minimum value above 1 being 1 + 1/1024. To minimize the accuracy loss while the parameters are being quantified from FP32 to FP16, the parameters should be small enough. However, in the case that λ starts with a small value, a model with a bunch of huge value parameters beyond FP16 might be obtained, especially in the first layer. To avoid this case, λ was set to 0.01 during the first 100 k iterations and then set to 0.001.

#### 3.2.2. Loss

A YOLOv3 (Farhadi and Redmon, 2018) object detector predicts bounding boxes using dimension clusters as prior boxes. For each bounding box, there are 4 corresponding predicted values, i.e., *t*_*x*_, *t*_*y*_, *t*_*w*_, and *t*_*h*_. When the center of the object is in the cell offset from the top left corner of the image by (*c*_*x*_, *c*_*y*_), and the prior box has the dimension o (*p*_*w*_, *p*_*h*_), then the prediction values correspond to


(2a) $$\begin{array}{l} b_{x}=\sigma \left(t_{x}\right)+c_{x} \end{array}$$

(2b) $$\begin{array}{l} b_{y}=\sigma \left(t_{y}\right)+c_{y} \end{array}$$

(2c) $$\begin{array}{l} b_{w}=p_{w}\cdot e^{t_{w}} \end{array}$$

(2d) $$\begin{array}{l} b_{h}=p_{h}\cdot e^{t_{h}} \end{array}$$

As in Figure 3:

**Figure 3:**
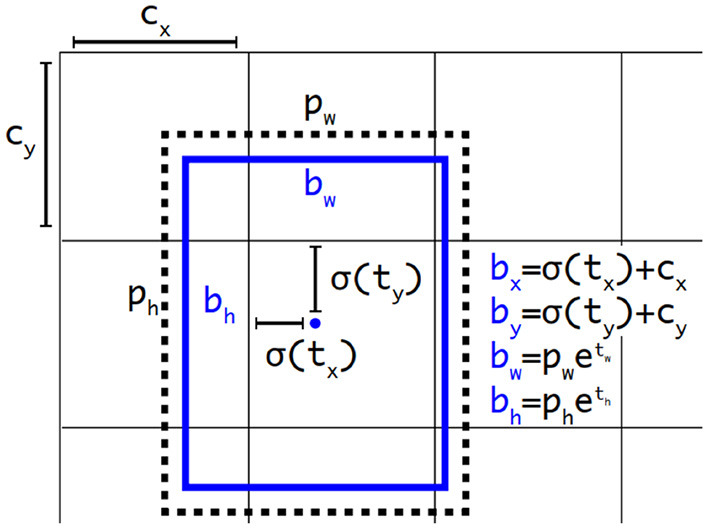
Bounding boxes with dimension priors and location prediction for YOLOv3.

For YOLOv3, a prediction loss comprises 3 parts, i.e., the object loss *L*_*obj*_, the classification loss: *L*_*cls*_, and the coordinate loss *L*_*box*_.


(3) $$\begin{array}{l} Loss=L_{obj}+L_{cls}+L_{box} \end{array}$$

Where


(4a) $$\begin{array}{l} L_{obj}=\lambda_{noobj}\overset{S^{2}}{\underset{i}{\sum }}\overset{B}{\underset{j}{\sum }}{𝟙}_{i,j}^{noobj}{\left(c_{i}-\hat{c_{i}}\right)}^{2}+\lambda_{obj}\overset{S^{2}}{\underset{i}{\sum }}\overset{B}{\underset{j}{\sum }}{𝟙}_{i,j}^{obj}{\left(c_{i}-\hat{c_{i}}\right)}^{2} \end{array}$$

(4b) $$\begin{array}{l} L_{cls}=\lambda_{cls}\overset{S^{2}}{\underset{i}{\sum }}\overset{B}{\underset{j}{\sum }}{𝟙}_{i,j}^{obj}\underset{c\in classes}{\sum }p_{i}\left(c\right)log\left(\hat{p_{i}}\left(c\right)\right) \end{array}$$

(4c) $$\begin{array}{l} L_{box}=\lambda_{box}\overset{S^{2}}{\underset{i}{\sum }}\overset{B}{\underset{j}{\sum }}{𝟙}_{i,j}^{obj}\left(2-w_{i}\times h_{i}\right) \end{array}$$

(4d) $$\begin{array}{l} \times \left[{\left(x_{i}-\hat{x_{i}}\right)}^{2}+{\left(y_{i}-\hat{y_{i}}\right)}^{2}+{\left(w_{i}-\hat{w_{i}}\right)}^{2}+{\left(h_{i}-\hat{h_{i}}\right)}^{2}\right] \end{array}$$

where *S* denotes the size of the feature map to be predicted, *B* represents the prior boxes count, ${𝟙}_{i,j}^{obj}$ refers to the fact that the *i*-th cell and the *j*-th prior box are responsible for one ground truth, and ${𝟙}_{i,j}^{noobj}$ refers to the opposite.

In this study, only palms need to be detected, therefore, it is always assumed that *L*_*cls*_ = 0. Moreover, Focal Loss (Lin et al., 2017) is used in *L*_*obj*_ to increase the recall rate *R* and suppress the erroneous recall rate *FP* :


(5) $$\begin{array}{l} L_{obj}=-\lambda_{obj}\overset{S^{2}}{\underset{i}{\sum }}\overset{B}{\underset{j}{\sum }}{𝟙}_{i,j}^{obj}\alpha {\left(1-c_{i}\right)}^{\gamma }\log\left(c_{i}\right) \end{array}$$

where the parameters of α and γ were set to 0.5 and 0.2, respectively. Focal Loss accelerates convergence during the training process because it gives much higher penalty weights to poor predictions. If a prediction value is close to the ground truth value, then it's much less important to keep minimizing the gap. As a result, the training process can pay more attention to poor predictions.

In terms of *L*_*box*_, CIOU Loss proposed in Zheng et al. (2020) is used just as Bochkovskiy et al. (2020) do, express as follows,


(6) $$\begin{array}{l} L_{box}=\lambda_{box}\overset{S^{2}}{\underset{i}{\sum }}\overset{B}{\underset{j}{\sum }}{𝟙}_{i,j}^{obj}\\ \left(1-{IoU}_{i}+\frac{{\left(x_{i}-\hat{x_{i}}\right)}^{2}+{\left(y_{i}-\hat{y_{i}}\right)}^{2}}{c_{i}^{2}}+\frac{v_{i}^{2}}{\left(1-{IoU}_{i}\right)+v_{i}}\right) \end{array}$$

Where $c_{i}^{2}$ is the area of the minimum box containing the prediction box and ground truth box.


(7) $$\begin{array}{l} v_{i}=\frac{4}{\pi }\left(\arctan\frac{\hat{w_{i}}}{\hat{h_{i}}}-\arctan\frac{w_{i}}{h_{i}}\right) \end{array}$$

The values of λ_*obj*_ and λ_*box*_ are set to 1 and 0.2, respectively, but when the model is hard to converge, λ_*box*_ can be adjusted according to the condition.

#### 3.2.3. Network Slimming

Studies demonstrated that accuracy can be improved by increasing the layers (deeper layers) (Simonyan and Zisserman, 2014) or the channels in layers (wider layers) (Howard et al., 2017). In this study, an initial network architecture wider and deeper enough was used, and the network was made to learn its structural sparsity. Besides, network slimming was used as well, which was introduced in the previous section. The slimming was performed on a well-trained network when the importance of the γ parameters in the BN layers was further learned. In addition, no regularization was imposed on the parameters in the convolutional layers, and the model was re-trained after pruning.

The training scheme in network slimming was similar to that of normal training, specifically, λ (the weight_decay value) started from 0.01 and then 0.001 after 100 k iterations.

#### 3.2.4. Background Training

As shown in Figure 1, in most areca plantations, the contrast between foreground and background was not very obvious. Almost all the images were green with the variation from light to dark green, except for some yellowish-brown spots which were the YLD diseased palm individuals.

All the palms in the dataset were labeled, and to increase the accuracy, the predictor was trained so that the background will not be predicted as objects. Those prior boxes not overlapped with any of the ground truths were defined as “background boxes.” During the training phase, in the case that a background box was predicted as an object, in another word, the confidence value was larger than the threshold value (e.g., 0.5), the predictor was punished. This extra work strengthened the predictor's ability to distinguish objects from the background, and decreased false-positive predictions.

### 3.3. Prior Boxes

Some studies involved the in-depth investigation of prior box selection in the YOLO model. It is empirically believed that some losses of accuracy were originated from the unequal distribution of the ground truth by anchors, in another word, one specific prior box in a cell response predicted more than the ground truth, so that during the training process, there is no way to learn all the ground truth. One solution to this problem is to avoid this conflict. For example, to use better designed prior boxes array or a bigger prior box collection. It is unnecessary to use more predictors for a light model (e.g., in Mazzia et al., 2020) if the backbone network has enough representational power, which is because that more predictors bring more computation complexity. In the study, the k-means was used to pick prior boxes for our model over the dataset, for example, in YOLOv3-Tiny, at first, *k* = 6 was set to get a box array of (23,23), (35,36), (48,49), (64,66), (90,91), and (147,157), which was referred to as "def-anchors" in the later section, with all the images normalized to (416,416). Since the smallest box is bigger than a high-resolution cell grid (16,16) in both width and height, another box array of (10,14), (27,23), (37,58), (75,64), (93,104), and (187,163) was used, which was referred to as “cust-anchors” to see what happens if there is one smaller prior box than the smaller cell grid. This study also used k = 8 to get another box array of (19,19), (27,29), (37,36), (43,48), (58,57), (71,75), (99,101), (158,169), referred to as “8-anchors.”

### 3.4. The Structure of YOLOv3-Tiny and Some Related Components

**Figure 4:**
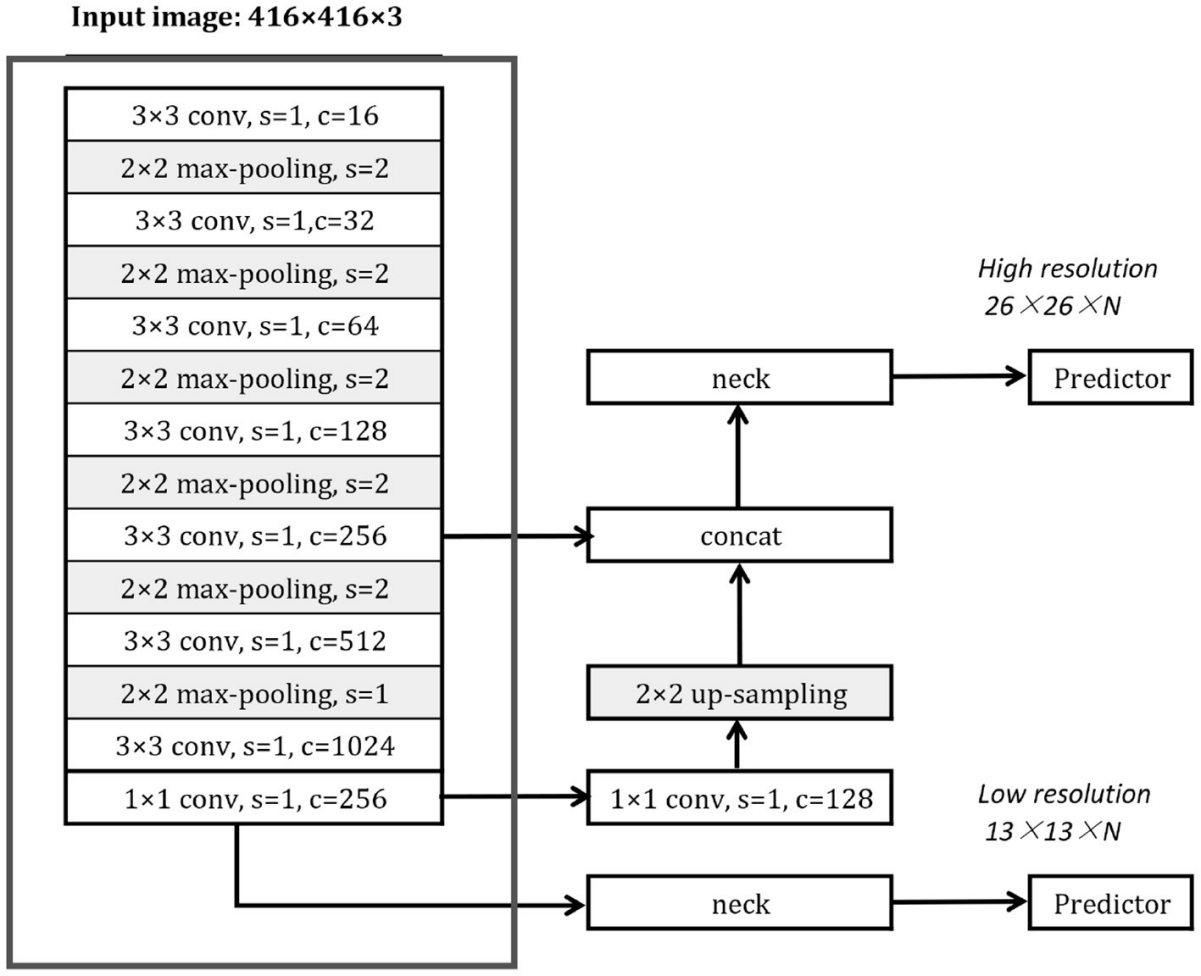
The YOLOv3-Tiny framework, s: stride, c: channel.

The basic YOLOv3-Tiny architecture is shown in Figure 4. As the one-stage object detector, it comprises a backbone network, one or more prediction heads, and corresponding necks. The backbone network of YOLOv3-Tiny is named *Darknet18*, which is framed by a red box. The YOLOv3-Tiny has 2 prediction heads in different scales and corresponding necks, which synthesizes and organizes high-level features of the input images.

**Figure 5:**
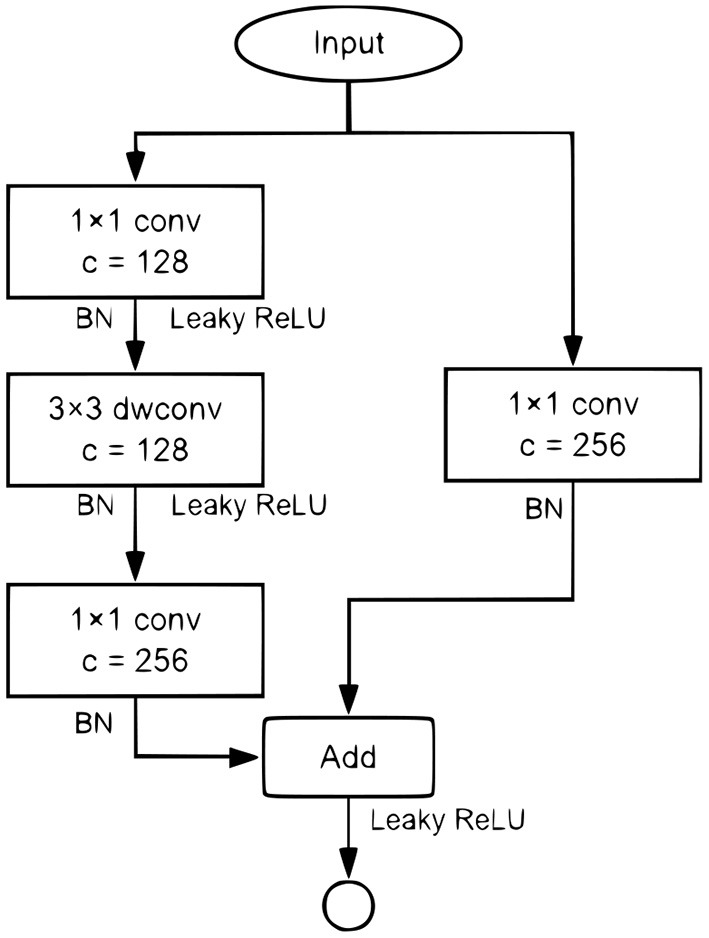
The ResBlock in PeeleNet.

The YOLOv3-Tiny uses an intuitive neck structure, which takes quite a lot of computation overhead. The ResBlock component proposed in PeleeNet (Wang et al., 2018), as in Figure 5, was used to compare with the original neck.

**Figure 6:**
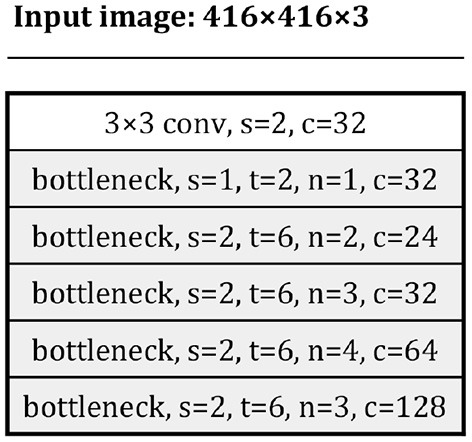
The compact MobileNet v2 backbone.

Some efficient backbone networks were evaluated, and it was found that the backbone networks proposed by MobileNet v2 and those proposed by ShuffleNet v2 showed time performance. In this study, they were also adapted for better performance. Figure 6 shows a compact version of MobileNet v2's backbone, and the bottleneck module is as defined in the original study.

**Figure 7:**
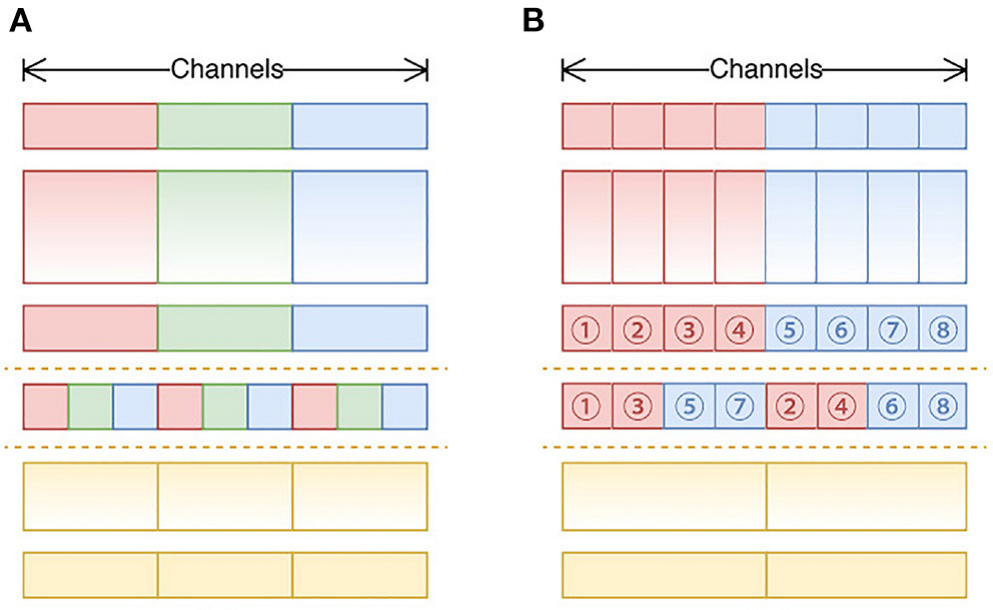
**(A)** The standard channel shuffle operation in ShuffleNet; **(B)** channel reorganization operation in our study.

**Figure 8:**
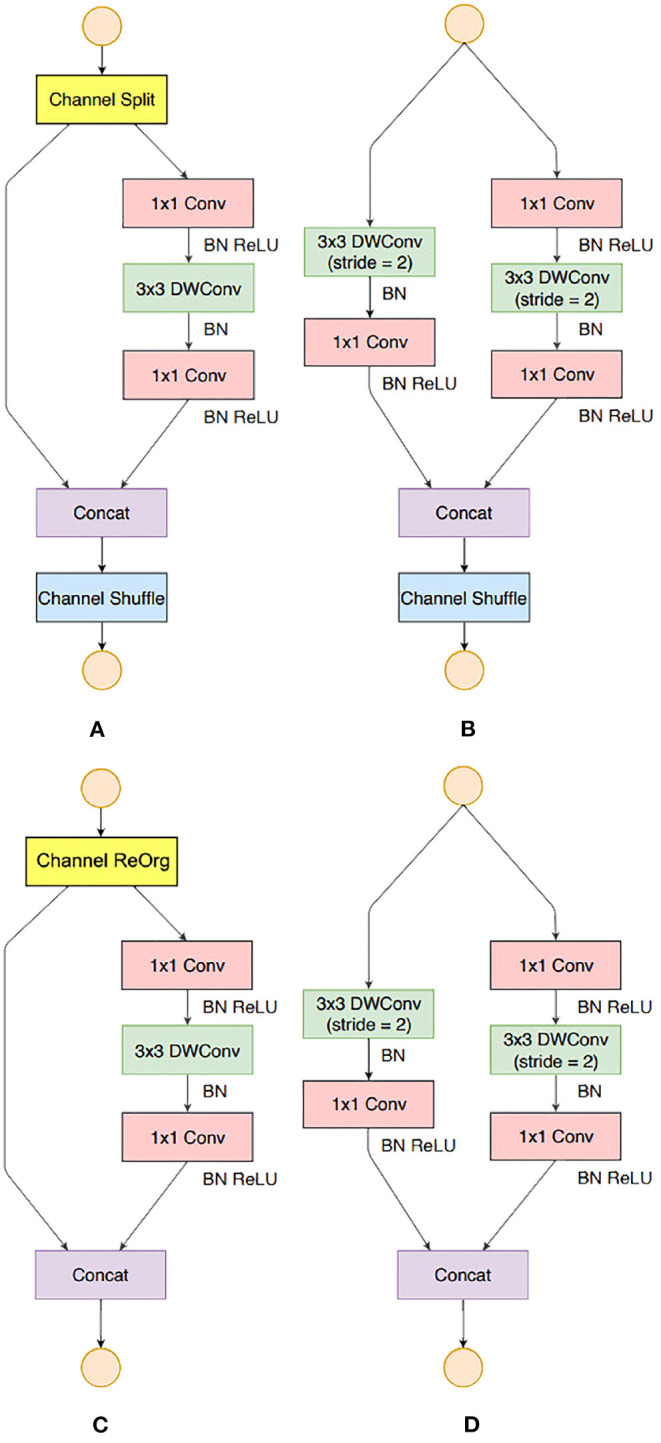
Building Blocks of ShuffleNet v2 and this study. **(A)** the basic ShuffleNet v2 unit; **(B)** the ShuffleNet v2 unit for spatial down sampling (2×); **(C)** our basic unit; **(D)** our unit for spatial down sampling (2×).

ShuffleNet v2 building blocks were used to build our backbone except for some small modifications. There were two reasons, the first is that the *Channel Shuffle* operation (as shown in Figure 7A) was not supported by the NCS2 hardware, therefore, *Channel Reorganization* was used to achieve the same or similar effect, (as shown in Figure 7B); Second, in this study, we found that in MobileNet v2 (as shown in Figures 8A,C), an activation layer followed a 3 × 3 depth-wise convolutional layer instead of a 1 × 1 one, and it showed higher accuracy (as shown in Figures 8B,D). This study compared the three cases as follows, 1) A 3 × 3 depth-wise convolutional layer followed by an activation layer without activation layer for the 1 × 1 convolutional layer; 2) No activation layer for 3 × 3 depth-wise convolutional layers, and a 1 × 1 convolutional layer is followed by an activation layer; 3) Both convolutional layers are followed by activation layers, with the details described in Section 4.

**Figure 9:**
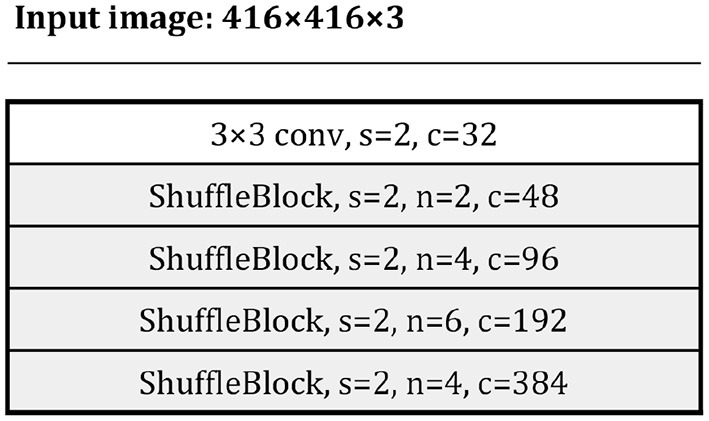
The ShuffleNet v2 backbone. Each line describes a sequence of one or more identical (modulo stride) layers, repeating for n times. All layers in the same sequence have the same number c of output channels. The first layer of each sequence has a stride s and all others use stride 1. All spatial convolutions use 3 × 3 kernels.

Figure 9 shows our backbone architecture.

### 3.5. Evaluation Metrics

**Figure 10:**
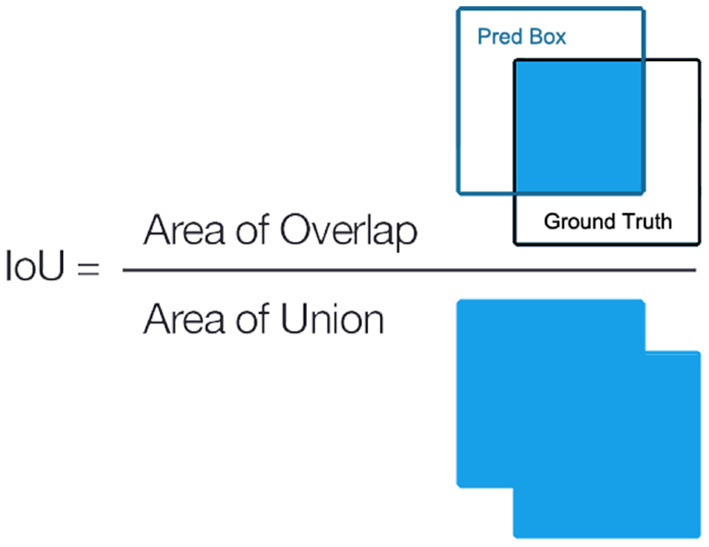
Definition of Intersection of Union (IoU).

The inference time on NCS2, Parameters Size (of the model), Billion FLoating Operations (BFLOPs), F1 score, and Intersection of Union (IoU) were applied to evaluate the detection performance. Inference time denotes the duration of detecting objects from one image which is a speed metric, BFLOPs which is a computational complexity metric, F1 score combines the performance evaluation of the recall and the precision of the detection, therefore, it is widely applied as the evaluation index in many previous studies of object detection when there is only one object category. The expression of the precision, recall, and F1 is expressed as follow:


(8a) $$\begin{array}{l} P=\frac{TP}{TP+FP} \end{array}$$

(8b) $$\begin{array}{l} R=\frac{TP}{TP+FN} \end{array}$$

(8c) $$\begin{array}{l} F_{1}=\frac{2\times P\times R}{P+R} \end{array}$$

where *P* denotes the precision, *R* refers to the recall, *TP* represents the true positives, *FP* is the false positives, and *FN* denotes the false negatives. The definition of IoU is shown in Figure 10, which measures the intersection area of the predicted object boundary box and the ground truth, thereby evaluating the location accuracy of the predicted boundary box of the prediction.

## 4. Results

In this section, a performance comparison was made among the vanilla YOLOv3-Tiny, the YOLOv3-Tiny with training improvement skills, the algorithm comprised of the Darknet18 backbone and the ResBlock neck, the algorithm comprised of efficient backbone and the ResBlock neck.

Based on the resulted data, we obtained Ag-YOLO.

### 4.1. Improving YOLOv3-Tiny Without Change to Structure

The Darknet18 backbone is very simple, which can be deemed as the simplest structure according to the principle proposed by VGG16. It is also a very good structure to inspect the performance data for NCS2. In this section, the impact of different prior boxes and background training was investigated.

Table 2 shows the accuracy incremental improvement obtained by background training and prior boxes selection.

**Table 2:** Accuracy incremental improvement.

**Model**	**Average**	**F1 Score**		**Parameters**	**BFLOPs**	**Inference**
	**IoU**	**IoU_0.5_**	**IoU_0.75_**	**Size**		**Time on NCS2**
Default-anchors-no-bg	0.8186	0.9236	0.7358	8.634 M	5.436	37.75 ms
Default-anchors-bg	0.8202	0.9250	0.7503	8.634 M	5.436	37.75 ms
Custom-anchors-bg	0.8170	0.9278	0.7422	8.634 M	5.436	37.75 ms
8-anchors-bg	**0.8266**	**0.9300**	**0.7685**	8.668 M	5.449	37.75 ms

*Bold values indicate the best performance*.

By training background and using more prior boxes, the accuracy was improved with a very small computational overhead, which was not reflected in the inference time on NCS2. The reason is that the hand-selected prior boxes failed to bring notable accuracy changes due to the fact that there were not enough small objects in the trained or tested images. What has to be pointed out is that the "Inference Time on NCS2" refers to the net computation time spent on the device, excluding the image decoding and data transfer.

### 4.2. Using the ResBlock Neck

The ResBlock component is proposed in PeleeNet (Wang et al., 2018). As shown in Figure 5.

Table 3 shows the performance improvement brought by the ResBlock neck. In this comparison, 8 prior boxes are used in predictors (4 by each).

**Table 3:** Accuracy improvement by ResBlock.

**Model**	**Average**	**F1 Score**		**Parameters**	**BFLOPs**	**Inference**
	**IoU**	**IoU_0.5_**	**IoU_0.75_**	**Size**		**Time on NCS2**
YOLOv3-Tiny built-in	**0.8266**	0.9300	0.7685	8.668 M	5.449	37.75 ms
ResBlock	0.8247	**0.9432**	**0.7777**	**6.848 M**	**4.109**	**27.42 ms**

*Bold values indicate the best performance*.

The F1 score in both **IoU**_**0.5**_ and **IoU**_**0.75**_ markedly improved at a cost of a slight drop in average IoU, which was acceptable. Moreover, the parameters were reduced from 8.668 million to 6.848 million, and BFLOPs were reduced from 5.449 to 4.109 billion, leading to an inference time shrink of more than 10 ms, i.e., about 27% of the original value.

### 4.3. Network Slimming

In the darknet source code,^4^ L2-norm regularization was imposed on the weights with λ = 0.0005. This value was too small, which leads to big parameters beyond FP16. Starting from λ = 0.01, this study found that in some channels, all the parameters tended to be zero, therefore, those channels were removed to reduce computation. However, by imposing L1-norm regularization on the γ parameters in batch normalization layers, a better result was achieved. This study pruned all the channels with |γ| < 0.5, and the pruned results were shown in Table 4.

**Table 4:** Performance data by pruning.

**Model**	**Average**	**F1 Score**		**Parameters**	**BFLOPs**	**Inference**
	**IoU**	**IoU_0.5_**	**IoU_0.75_**	**Size**		**Time on NCS2**
ResBlock	0.8247	0.9432	0.7777	6.848 M	4.109	27.42 ms
Pruned	0.8237	0.9433	0.7625	6.548 M	3.949	27.33 ms

Network slimming failed to bring notable performance improvement in terms of inference time, on the contrary, it induced a little degradation to IoU and F1 scores in this experiment. However, in some architectures, it generated a smaller model.

### 4.4. Using Efficient Backbones

#### 4.4.1. SqueezeNet

Table 5 shows the performance variation while Darknet18 was replaced by SqueezeNet. SqueezeNet achieved better performance, e.g., better IoU and fewer parameters. However, it doubled computation complexity and increased the inference time.

**Table 5:** Performance data of SqueezeNet.

**Model**	**Average**	**F1 Score**		**Parameters**	**BFLOPs**	**Inference**
	**IoU**	**IoU_0.5_**	**IoU_0.75_**	**Size**		**Time on NCS2**
Default-anchors-no-bg	0.8186	0.9236	0.7358	8.634 M	5.436	37.75 ms
SqueezeNet	0.8346	0.9304	0.7860	1.186 M	5.176	56.67 ms

Table 6 shows its performance. With the bottleneck microstructure, improvements were made in terms of IoU, F1 score, and BFLOP. However, the inference time was much shorter (about 2.5 times that by Darknet18).

**Table 6:** Performance data of MobileNet v2.

**Model**	**Average**	**F1 Score**		**Parameters**	**BFLOPs**	**Inference**
	**IoU**	**IoU_0.5_**	**IoU_0.75_**	**Size**		**Time on NCS2**
Default-anchors-no-bg	0.8186	0.9236	0.7358	8.634 M	5.436	37.75 ms
MobileNet v2	0.8443	0.9592	0.8240	1.082 M	2.095	65.32 ms

Table 7 shows the results. The second one has the best performance, and for the third one, more non-linearity leads to worse performance. Combined with a modified version of ShuffleNet-v2 backbone, a ResBlock neck, and a YOLOv3 head, a new YOLO framework were proposed. We temporarily named it *Ag-YOLO* because it could be used for agricultural purposes.

**Table 7:** Performance data of ShuffleNet v2.

**Model**	**Average**	**F1 Score**		**Parameters**	**BFLOPs**	**Inference**
	**IoU**	**IoU_0.5_**	**IoU_0.75_**	**Size**		**Time on NCS2**
Default-anchors-no-bg	0.8186	0.9236	0.7358	8.634 M	5.436	37.75 ms
ShuffleNet v2(1)	**0.8349**	0.9448	**0.7893**	813 K	1.033	26.23 ms
ShuffleNet v2(2)	0.8278	**0.9513**	0.7668	**711 K**	**0.985**	**25.96 ms**
ShuffleNet v2(3)	0.8178	0.9404	0.7394	878 K	1.071	27.60 ms

*Bold values indicate the best performance*.

### 4.5. Models Tested on NCS2

All models were converted to OpenVINO-version and tested on an NCS2 device. The host was a Windows 10 laptop and the data was transferred *via* USB3 protocol also supported by the RPi 4 computer. Due to the data precision loss, performance degradation occurred for all models. As seen from Table 8, the Ag-YOLO improved the original YOLOv3-Tiny version significantly in terms of both speed (about 6 frames more in a second) and accuracy (about 0.2 increase in F1 score). The model using a compact MobileNet v2 backbone surpassed our model a little in terms of F1 score and IoU, however, it takes double times to run.

**Table 8:** Models tested on NCS2.

**Model**	**Features**				**Backbone**	**fps**	**F1 Score**	**IoU**
	**CIoU Loss**	**BG**	**ResBlock**	**Pruned**				
1 (YOLOv3-Tiny)					Darknet18	20.7	0.9160	0.6959
2	✓				Darknet18	20.7	0.9276	0.7148
3	✓	✓			Darknet18	20.7	0.9302	0.7253
4	✓	✓	✓		Darknet18	26.2	0.9223	0.7209
5	✓	✓	✓	✓	Darknet18	26.3	0.9209	0.7108
6	✓	✓	✓	✓	PeleeNet	14.6	0.9211	0.7352
7	✓	✓	✓	✓	Compact MobileNet v2	13.0	0.9364	0.7410
8 (Ag-YOLO)	✓	✓	✓	✓	ShuffleNet v2 derived	**26.9**	0.9361	0.7395

*IoU = 0.5, input dimensions: 416 × 416, confidence threshold = 0.4, and non-maximum-supress threshold = 0.5. Bold value indicates the best performance*.

When the input dimension was set to 352 ×352, Ag-YOLO achieved the speed of 36.5 fps with an F1 score of 0.9205 and IoU of 0.708 on NCS2, while YOLOv3-Tiny achieved similar accuracy at the speed of 18.1 fps with an input dimension of 448 ×448. Based on these data, Ag-YOLO is two times faster than YOLOv3-Tiny.

**Figure 11:**
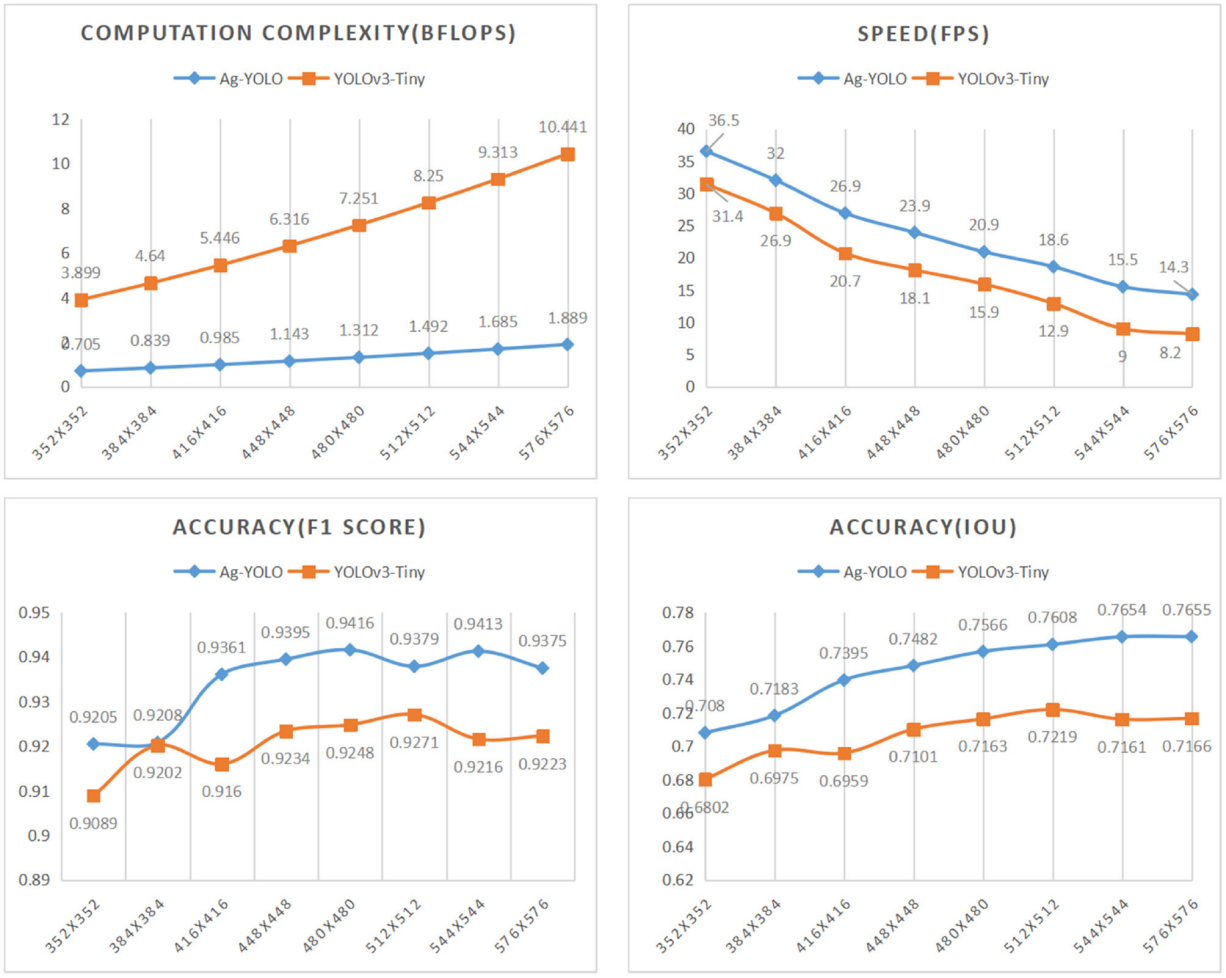
Ag-YOLO vs. YOLOv3-Tiny under different input dimensions.

**Figure 12:**
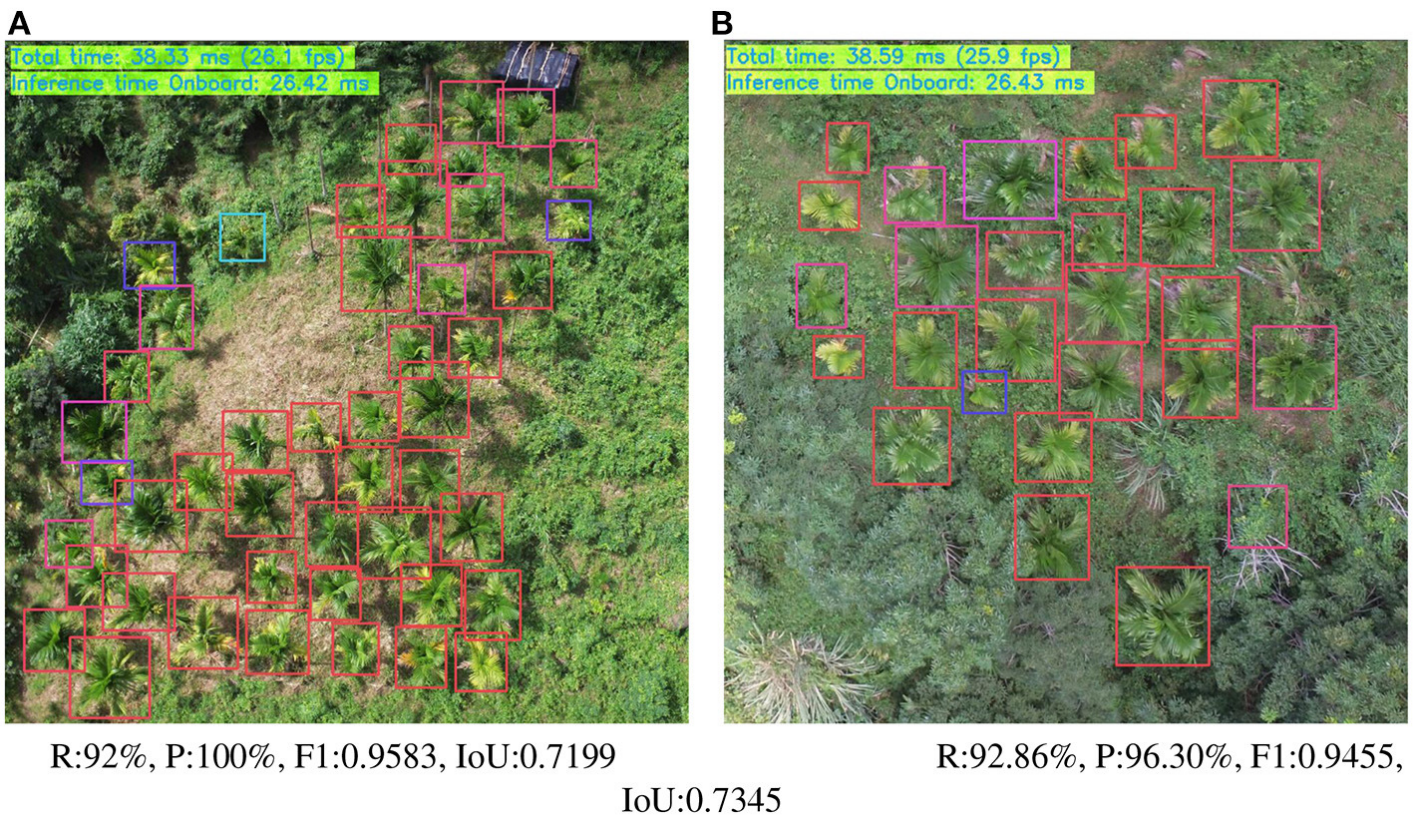
Ag-YOLO run on NCS2 (Input dimension: 416 × 416). Images were taken by a UAV, different colors of the predicted square imply different confidence values, blue is low, and red is high. **(A)** R:92%, P:100%, F1:0.9583, IoU:0.7199. **(B)** R:92.86%, P:96.30%, F1:0.9455, IoU:0.7345.

**Different input dimensions:** Performance of a model is also affected by the input dimension. As in the training phase, the input dimensions had been changed to 352 × 352, 384 × 384, 416 × 416, 448 × 448, 480 × 480, 512 × 512, 544 × 544, 576 × 576. Figure 11 shows performance trends of Ag-YOLO and YOLOv3-Tiny. Figure 12 is an example of the NCS2 output.

## 5. Discussion

Abdulridha et al. (2019) applied a hyperspectral camera for the detection of citrus canker disease in citrus plantations. Modica et al. (2020) used UAV multi-spectral imagery to monitor the vigor in heterogeneous citrus and olive orchards. Ye et al. (2020) identified Fusarium wilt in bananas using supervised classification algorithms with UAV-based multi-spectral imagery. Those camera systems are characterized by expensiveness, difficulty in operation, relatively large size, and susceptibility to crash situations compared with RGB cameras.

The developed software, presented in this study, is specially adapted for use in embedded RGB-camera systems. With the increasing availability of UAVs that can spray pesticides, the algorithm can contribute to performing selective spraying. Therefore, the pesticides could be saved, thereby reducing the environmental impact and the economical costs of the farmer. In particular, cheap technology is necessary for the wide use of target-orientated selective spraying. Additionally, a cheap RGB-camera-controlled UAV spraying should also be affordable for small farmers.

The source code of this study is available at https://github.com/rossqin/RQNet, which can be used as a reference for the beginning researchers to develop their real-life AI applications instead of pursuing higher performance with new algorithms and the ever-increasing demand for higher computational power and memory requirements. In a specific agricultural CV task, for instance, object detection, the object category is usually one or few, therefore, it is possible to use a small and efficient DNN-based model to achieve a good result. This is proved in this study by exploring the YOLOv3-Tiny architecture and replacing the neck and backbone with different state-of-art efficient DNNs, such as SqueezeNet, MobileNet v2, and ShuffleNet v2. This study also uses network slimming to compress the models to obtain smaller models. In the meantime, this study trained all the models on a laptop and tested them on a low-cost hardware accelerator, i.e, the Intel NCS2. Our architecture, the Ag-YOLO, is comprised of a ShuffleNet v2-derived backbone, a ResBlock neck, and a YOLOv3 head, with only 813k parameters and 1.033 billion FLOPs, which is only 9.4 and 19% of the Darknet18 version, respectively. However, it brings better accuracy and inference time performance on the resource-constraint hardware NCS2, achieving a speed of 36.5 fps. Because a camera usually takes video at the frame rate of 24 fps, this is a REAL-TIME object detector. On the other hand, a compact version of the MobileNet v2 backbone leads to a better accuracy performance, although it takes more than twice BFLOPs and inference time. In a UAV or UGV auto-pilot use case, the host usually moves quite slow, and there is no need to process each frame from the onboard camera. For the tasks that emphasize accuracy, the compact version of the MobileNet v2 backbone presents a better option for Ag-YOLO. To obtain better accuracy, redundant information between successive frames can be utilized, as shown in Bozek et al. (2018).

## 6. Conclusion

This study decomposes the “YOLOv3-Tiny” into a backbone network, one or more necks, and corresponding heads. The backbone network extracts the features from an image, the necks synthesize the features that backbone network outputs, and the heads decode the information as required. This work improves YOLOv3-Tiny by replacing a more efficient backbone and better neck, in addition, we adopt some “Freebies” and “Back-of-Specials” such as CIOU Loss and more prior boxes in heads. Our work demonstrates that, a DNN-based CV algorithm can be implemented on resource-constraint device to deal with real-life PA challenge, even with the most costefficient embedded AI device, e.g., the NCS2. In addition, our Ag-YOLO can achieve 36.5 fps with satisfying accuracy. The accuracy of Ag-YOLO is always higher than that of YOLOv3-Tiny in different input dimensions, and the highest accuracy of Ag-YOLO reaches 0.7655. This experiment also demonstrated that a MobileNetv2-derived backbone showed better representational power, and a ShuffleNetv2-like backbone runs faster at the cost of a little accuracy degradation. Besides, both of them are superior in terms of computation intensity and memory usage. With this work, including the open-source toolset, it should be very easy to make their legacy agricultural machinery intelligent by using an onboard camera and an edge computing device.

## 7. Future Work

The proposed model Ag-YOLO has been proved to be competent in extending a UAV with little overhead, from cost to energy. The CV will be integrated into a practical spraying UAV *via* the Mavlink protocol to deal with the challenges in areca protection.

## Data Availability Statement

The datasets presented in this study can be found in online repositories. The names of the repository/repositories and accession number(s) can be found in the article/supplementary material.

## Author Contributions

WW: conceptualization. ZQ: methodology, software, writing—original draft preparation, data curation, and formal analysis. K-HD, ZQ, LG, and ZC: writing—review and editing. WW and LG: project administration. LG: funding acquisition. ZC: proofread. All authors have read and agreed to the published version of the manuscript.

## Funding

This work was funded by National Natural Science Foundation of China (Grant No. 31860180), Major Science and Technology Program of Inner Mongolia Autonomous Region (Grant No. ZD20190039), Natural Science Foundation of Xinjiang Uygur Autonomous Region (Grant No. 2018D01A20), and Science and Technology Project of Inner Mongolia Autonomous Region (Grant No. 2021GG0341).

## Conflict of Interest

The authors declare that the research was conducted in the absence of any commercial or financial relationships that could be construed as a potential conflict of interest.

## Publisher's Note

All claims expressed in this article are solely those of the authors and do not necessarily represent those of their affiliated organizations, or those of the publisher, the editors and the reviewers. Any product that may be evaluated in this article, or claim that may be made by its manufacturer, is not guaranteed or endorsed by the publisher.

## Footnotes

^1^ https://en.wikipedia.org/wiki/Sanya

^2^ https://www.dji.com/cn/phantom-4-rtk?site=brandsite&from=nav

^3^https://github.com/tzutalin/labelImg or https://pypi.org/project/labelImg/

^4^ https://pjreddie.com/darknet/
